# Lanolin

**Published:** 1890-08-09

**Authors:** 


### LANOLIN.

Lanolin differs from other fats in having for its base cholestearin or isocholestearin in place of glycerine. All animals and some plants have these protective fats on their surface, serving as a varnish to resist atmospheric and other external influences. In animals the base is always cholestearin, but in plants this is conjoined with waxy bodies, as in the fruit of plums and the bloom on lupins, &c. The quantity depends on the climate and other circumstances; thus the wool of some European sheep contains little lanolin, whereas it is so abundant in that of Australian sheep, as to be forced out by simple pressure of the fleece. It is formed in the cells of the epithelium without the aid of glands, and exists in those of the human skin as may be shown by Liebermann's test for cholestearin: hence its value as an emollient. The *vernix caseosa* is largely composed of lanolin, with the keratin and debris of the cast off epithelium.

